# Roles of Chlorogenic Acid on Regulating Glucose and Lipids Metabolism: A Review

**DOI:** 10.1155/2013/801457

**Published:** 2013-08-25

**Authors:** Shengxi Meng, Jianmei Cao, Qin Feng, Jinghua Peng, Yiyang Hu

**Affiliations:** ^1^Institute of Liver Diseases, Shuguang Hospital Affiliated to Shanghai University of Traditional Chinese Medicine, 528 Zhangheng Road, Pudong, Shang hai 201203, China; ^2^Shanghai University of Traditional Chinese Medicine, 1200 Cailun Road, Pudong, Shanghai 201203, China; ^3^E-Institute of Traditional Chinese Internal Medicine of Shanghai Municipal Education Commission, 1200 Cailun Road, Pudong, Shanghai 201203, China

## Abstract

Intracellular glucose and lipid metabolic homeostasis is vital for maintaining basic life activities of a cell or an organism. Glucose and lipid metabolic disorders are closely related with the occurrence and progression of diabetes, obesity, hepatic steatosis, cardiovascular disease, and cancer. Chlorogenic acid (CGA), one of the most abundant polyphenol compounds in the human diet, is a group of phenolic secondary metabolites produced by certain plant species and is an important component of coffee. Accumulating evidence has demonstrated that CGA exerts many biological properties, including antibacterial, antioxidant, and anticarcinogenic activities. Recently, the roles and applications of CGA, particularly in relation to glucose and lipid metabolism, have been highlighted. This review addresses current studies investigating the roles of CGA in glucose and lipid metabolism.

## 1. Introduction

Intracellular glucose and lipid metabolic homeostasis is very vital for maintaining the basic life activities of a cell or an organism. In terms of cytology, intracellular glucose and lipid metabolic disorders are the basis of a variety of metabolic diseases. Glucose and lipid metabolic disorders are closely related with the occurrence and progression of diabetes, obesity, hepatic steatosis, cardiovascular disease, and cancer [1]. The complications of glucose and lipid metabolic disorders will impose a significant burden on health care systems all over the world. However, medical therapeutic options are not only limited, but also associated with unwanted side effects [2–4]. Therapies with novel mechanisms of action to combat glucose and lipid metabolic disorders would therefore have significant medical and economic impacts.

**Figure 1:**
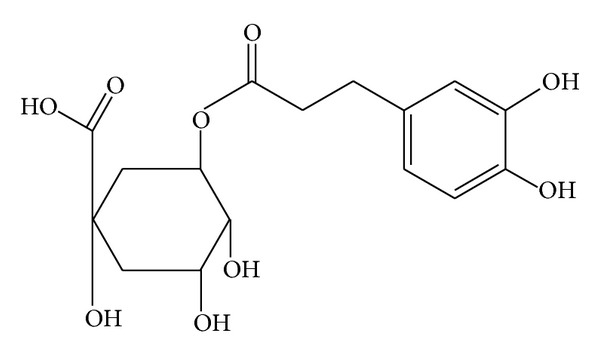
Chemical structure of chlorogenic acid (CGA).

**Figure 2:**
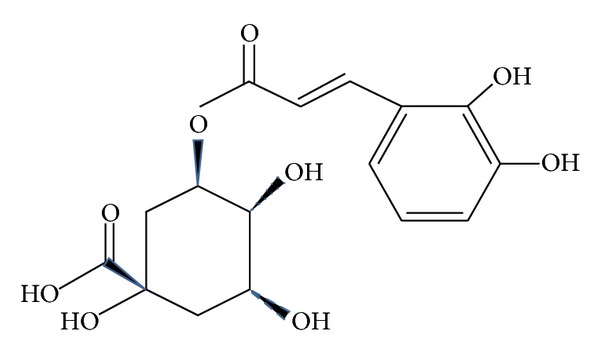
Chemical structure of 5-O-cafeoylquinic acid (chlorogenic acid).

Chlorogenic acid (CGA) (Figure 1), one of the most abundant polyphenol compounds in the human diet, is a group of phenolic secondary metabolites produced by certain plant species and an important component of coffee. It has been reported that coffee had the highest concentration of polyphenols among the beverages analyzed [5, 6]. The major polyphenol in coffee is CGA. Chlorogenic acid (CGA) is an ester formed from cinnamic acids and quinic acid and is also known as 5-O-caffeoylquinic acid (5-CQA) (IUPAC numbering) or 3-CQA (pre-IUPAC numbering) [7]. The most common form of CGA is 5-caffeoylquinic acid (5-CQA) (Figure 2). Accumulating evidence has demonstrated that CGA exhibits many biological properties, including antibacterial, antioxidant, and anticarcinogenic activities, particularly hypoglycemic and hypolipidemic effects [8–14]. CGA has been recently claimed to modulate glucose and lipid metabolism *in vivo* in both healthy and genetically metabolic disordered conditions [14–16].

Recently, the roles and the applications of CGA, particularly in relation to glucose and lipid metabolism, have been highlighted in both biological and medical fields [17–21]. It will possibly, we think, become a research focus or a trend of medicine and pharmacology in the near future. A review of the roles and applications of CGA in glucose and lipid metabolism is consequently urgent and vital to assist in further research.

## 2. Effects on Glucose Metabolism

### 2.1. Hypoglycemic and Antidiabetic Effect

 Regular consumption of coffee has been associated with a lower risk of type 2 diabetes mellitus (T2DM), and this has been replicated across sexes, geographical locations, and obesity levels [22–28]. CGA is major bioactive compound in coffee that may provide health benefits. For example, it is reported that daily consumption of 3 to 4 cups of decaffeinated coffee containing high contents of CGA significantly reduced the risk for T2DM by 30% [29].

Chlorogenic acid (CGA) is a novel insulin sensitizer that potentiates insulin action similar to the therapeutic action of metformin [30]. Chlorogenic acid (CGA) at a dose of 5 mg/kg body weight exerts antidiabetic potential in streptozotocin (STZ) (45 mg/kg b.w.) nicotinamide induced diabetic rats [31–33].

Bassoli et al. (2008) analysed the effects of CGA on hepatic glucose output, blood glucose levels, and glucose tolerance. It was found that CGA did promote a significant reduction in the plasma glucose peak in the oral glucose tolerance test, most likely by attenuating intestinal glucose absorption, indicating a possible role for CGA as a glycaemic index lowering agent and highlighting it as a compound of interest for reducing the risk of developing T2DM [12].

CGA exerts its antidiabetic effects on stimulating glucose uptake in both insulin-sensitive and insulin-resistant adipocytes. The potency of CGA to stimulate 2-[N-(7-nitrobenz-2-oxa-1,3-diazol-4-yl)amino]-2-deoxy-d-glucose(2-NBDG) uptake was comparable to the antidiabetic drug rosiglitazone [34]. Moreover, CGA, unlike thiazolidinedione (TZD) or insulin, does not induce obesity or other side effects.

It was reported that CGA ingestion significantly reduced early fasting glucose and insulin responses in overweight men during an OGTT [35]. Clinical trials have also testified that CGA is able to lower the glycemic impact of foods and chronically lower background blood glucose levels of T2DM [36]. 

### 2.2. Stimulation of Insulin Secretion

 CGA has been described as a potential antidiabetic agent. Using *in vitro* studies, Tousch et al. [37] (2008) reported that CGA increased glucose uptake in L6 muscular cells, an effect only observed in the presence of stimulating concentrations of insulin. In addition it was found that CGA stimulates insulin secretion from the INS-1E insulin-secreting cell line and rat islets of Langerhans. Clinical trials have also testified that CGA in coffee is able to modulate glucose uptake and gastrointestinal hormone and insulin secretion in humans [38].

### 2.3. Improving Glucose Tolerance and Insulin Resistance

 Insulin resistance is a major obstacle in the diabetes treatment and is often accompanied by hyperglycemia, hyperinsulinemia, and hyperlipaemia in obesity-induced type 2 diabetic patients, which is also regarded as one of the risk factors leading to a series of complications, such as nephropathy, retinopathy, myocardial infarction, and neuropathy [39]. CGA has been shown to act as an active principle in glucose metabolism regulation [40, 41]. CGA is capable of improving glucose tolerance and insulin resistance in obese (*fa*/*fa*) Zucker rats, suggesting that CGA may be a promising candidate for the development of antidiabetic agents [13]. Liang et al. [42] also found that, compared to model group (mice were administrated with high-fat emulsion by gastric perfusion), CGA interference group (mice were administrated with high-fat emulsion and CGA (20 mg·kg^−1^ body weight)) had better glucose tolerance, higher insulin sensitivity index (ISI), and lower HOMA-IR index. And the contents of TG, TC, and LDL-C in serum were decreased in the CGA interference group.

## 3. Effects on Lipid Metabolism

### 3.1. Lowering Serum and Hepatic CG and TG Levels

 Hypercholesterolemia is a major risk factor for the development of cardiovascular disease and nonalcoholic fatty liver disease. CGA are hypoglycemic agents and may affect lipid metabolism. Rodriguez de Sotillo and Hadley [14] investigated the effects of CGA *in vivo*, by using obese, hyperlipidemic, and insulin resistant (*fa*/*fa*) Zucker rats. The authors reported that CGA did not promote sustained hypoglycemia, but significantly lowered the postprandial peak response to a glucose challenge when compared to the same group of rats before CGA treatment. In CGA-treated rats, fasting plasma cholesterol and triacylglycerol concentrations significantly decreased by 44% and 58%, respectively, as did liver triacylglycerols concentrations (24%). There were no statistical differences (*P* > 0.05) in adipose triacylglycerol concentrations. Significant differences (*P* < 0.05) in the plasma, liver, and spleen concentration of selected minerals were found in CGA-treated rats. This study suggested that *in vivo* CGA improves glucose tolerance, decreases various plasma and liver lipids, and improves mineral pool distribution.

### 3.2. Reducing LDL Oxidation Susceptibility and Decreasing LDL-Cholesterol and MDA Levels

 It is currently believed that oxidative modification of low-density lipoproteins (LDL) by free radicals is a key early event in the pathogenesis of atherosclerosis. The rapid uptake of oxidatively modified LDL via a scavenger receptor leads to the formation of foam cells. Oxidized LDL also has a number of other atherogenic properties [43]. Chlorogenic acid may favorably affect cardiovascular risk status by modestly reducing LDL oxidation susceptibility and decreasing LDL-cholesterol and malondialdehyde (MDA) levels. Chlorogenic acid, active compound in coffee, inhibits oxidation of LDL *in vitro* and may therefore protect against cardiovascular disease [44, 45].

### 3.3. Inhibiting Fat Absorption and Activating Fat Metabolism in the Liver

 Shimoda et al. [46] (2006) testified that CGA, caffeine, and other polyphenolic compounds in green coffee bean extract (GCBE) act to suppress body weight gain and visceral fat accumulation in mice. The authors reported that CGA is possibly effective against weight gain and fat accumulation by inhibition of fat absorption and activation of fat metabolism in the liver. And oral administration of CGA (30 and 60 mg/kg/day) for 14 days dramatically reduced the level of hepatic TG in mice. The suppressive effect of CGA on hepatic TG accumulation was more potent than that of GCBE.

### 3.4. Improvement of Obesity-Related Hormones Levels

 Cho et al. [47] (2010) investigated the efficacy of CGA on altering body fat in high-fat diet (37% calories from fat) induced obese mice compared to caffeic acid. The authors found that CGA significantly lowered body weight, visceral fat mass and plasma leptin, and insulin levels compared to the high-fat control group. CGA also lowered triglyceride (in plasma, liver, and heart) and cholesterol (in plasma, adipose tissue, and heart) concentrations. Chlorogenic acid significantly inhibited fatty acid synthase, 3-hydroxy-3-methylglutaryl CoA reductase, and acyl-CoA cholesterol acyltransferase activities, while they increased fatty acid beta-oxidation activity and peroxisome proliferator-activated receptors alpha expression in the liver compared to the high-fat group. The authors' results suggested that CGA can improve body weight, lipid metabolism, and obesity-related hormones levels in high-fat fed mice.

### 3.5. Alterations in Lipids, Lipoproteins, and Enzymes Involved in Lipid Metabolism

 Diabetes mellitus is associated with dyslipidemia which is a significant risk factor for cardiovascular complications. Karthikesan et al. [48] evaluated the effects of CGA on alterations in lipids, lipoproteins, and enzymes involved in lipid metabolism in STZ-nicotinamide-(NA-) induced T2DM rats. The authors found that there was a significant increase in the concentrations of plasma and tissue (liver and kidney) lipids, cholesterol, triglycerides (TGs), free fatty acids (FFAs) and phospholipids (PLs), and low density and very low-density lipoproteins (LDL and VLDL), respectively, and a decrease in the concentration of high-density lipoproteins (HDL) in STZ administered diabetic rats. In addition, the activity of 3-hydroxy 3-methylglutaryl coenzyme A (HMG-CoA) reductase increased significantly in the liver and kidney whereas the activities of lipoprotein lipase (LPL) and lecithin cholesterol acyl transferase (LCAT) were decreased significantly in the plasma of diabetic rats. Administration of CGA remarkably reduced the STZ-induced changes in lipids, lipoproteins, and lipid metabolising enzymes in diabetic rats. The author's results indicate that CGA can potentially ameliorate lipid abnormalities in experimental T2DM. 

### 3.6. Inhibiting Lipids' Absorption and Transformation, Inhibiting Cholesterol's Intestinal Absorption and Hepatic Biosynthesis

 Li et al. [49] (2012) observed the effects of CGA on key enzyme activities in lipid metabolism and explored its antihyperlipidemia mechanism. The authors studied the lipid-lowering effect and mechanism of CGA by observing the influence on the formation of cholesterol micelles and on the inhibition of 3-hydroxy-3-methylglutaryl-CoA (HMG-CoA) reductase from normal pig liver as well as pancreatic lipase *in vitro*. The authors found that CGA has strong inhibitory effects on cholesterol micelles formation and has stronger inhibitory potency on HMG-CoA reductase than simvastatin. In addition, CGA also has a stronger inhibition on the activity of pancreatic lipase. The mechanism of CGA in reducing blood lipids was most likely associated with the inhibition of absorption and transformation of lipids and with the inhibition of intestinal absorption and hepatic biosynthesis of cholesterol.

### 3.7. Improvement of Antioxidant Activities

 Wang et al. [50] (2012) investigated the effect of CGA on lipid metabolism of hyperlipidemia mice. It was found that the contents of serum TC, TG, LDL-C levels, and liver TC were significantly lower. Furthermore, malondialdehyde (MDA) contents in serum and liver were decreased, and activities of antioxidant enzymes were increased. Arteriosclerosis index (AI) was also lower than that of the model group. The results indicated that CGA could effectively reduce the blood and liver lipid accumulation and regulate lipid metabolism by improving their antioxidant activities. Furthermore, the group at the dose of 50 mg/kg CGA showed the best effect among all groups.

## 4. Mechanism of Action

### 4.1. Improvement of Cellular Mechanisms

*In vivo* studies have confirmed that CGA can improve glucose tolerance and mineral pool distribution in obese Zucker rats [14]. The significant decrease in postprandial blood glucose concentrations may be attributed to an improved sensitivity to insulin [51]. Impaired glucose tolerance and insulin resistance have been associated with differences in the hepatic mRNA expression of the spliced variants of the insulin receptor at exon 11. Spliced variants of the insulin receptor have not been studied in obese Zucker rats. Thus, Rodriguez de Sotillo et al. [15] (2006) studied the *in vivo* effect of CGA on plasma insulin concentrations in a glucose tolerance test. It was found that in the CGA-treated group, areas under the curve (AUC) for blood glucose and plasma insulin improved, and the protein and DNA concentrations in the liver increased. There were no significant differences between groups for the hepatic G-6-Pase activity. The insulin receptor exon 11 (+) and the exon 11 (−) variants were expressed in the liver of Zucker (*fa*/*fa*) rats without significant changes. It is consequently suggested that CGA may improve some cellular mechanisms that are stimulated by insulin.

### 4.2. Inhibition of the Activity of *α*-Glucosidase

 Zheng et al. [52] (2007) examined the inhibitory effect of CGA on the postprandial blood glucose concentration in rats. It was reported that CGA inhibited the activities of *α*-amylase and *α*-glucosidase and reduced the postprandial blood glucose concentration. Chlorogenic acid (CGA), as well as acarbose, strongly inhibited the activity of *α*-glucosidase and reduced the postprandial blood glucose concentration. It was reported that CGA suppresses postprandial hyperglycemia by inhibiting *α*-glucosidase and that its action resembles that of currently available *α*-glucosidase inhibitors such as acarbose, miglitol, and voglibose [53, 54]. Matsui et al. described that CGA inhibits rat intestinal *α*-glucosidase in a noncompetitive manner [55]. 

In particular, CGA has been implicated to be responsible for anti-hyperglycemic effects in humans [56]. McCarty [57] had reported that the consumption of coffee by humans reduced the rise of plasma glucose concentrations in a tolerance test. It suggested that CGA may exert an antagonistic effect on glucose transport. Experiments with everted gut sac have showed that CGA inhibits the uptake of glucose from the rat intestine. Their results suggested that CGA may inhibit *α*-glucosidase by the attenuation of glucose transport in a synergistic manner.

### 4.3. Alteration of GIP Concentrations

 Chlorogenic acid (CGA) may inhibit intestinal glucose uptake *in vitro*. Furthermore, CGA is thought to stimulate the secretion of glucagon-like peptide-1 (GLP-1), which is known to have a beneficial effect on the response to glucose in pancreatic beta cells [58]. To elucidate the mechanisms by which CGA acts to mediate blood glucose response *in vivo*, Tunnicliffe et al. [59] (2011) investigated Sprague-Dawley rats that were catheterized and gavage-fed a standardized meal administered with or without CGA in a randomized crossover design separated by a 3-day washout period. It was found that the total area under the curve (AUC) for blood glucose was significantly attenuated in rats fed with CGA (*P* < 0.05). In contrast, no differences in plasma insulin nor nonesterified fatty acids, and gastric emptying were observed. Plasma glucose-dependent insulinotropic peptide (GIP) response was blunted in rats fed with CGA, with a lower peak concentration and AUC up to 180 min postprandially (*P* < 0.05). There were no changes in GLP-1 secretion in either the *in vivo* or *in vitro* studies. It was demonstrated that CGA treatment resulted in beneficial effects on blood glucose response, with alterations seen in GIP concentrations. In view of the widespread consumption and availability of coffee, CGA may be a viable preventative tool for T2DM. 

### 4.4. Activation of AMPK

 AMP activated protein kinase (AMPK) is a master sensor and regulator of cellular energy balance [60]. It is activated by various pharmacological, pathological, and metabolic stressors such as metformin, thiazolidinediones, hypoxia and exercise. Activation of AMPK leads to translocation of GLUT4 from intracellular membranes to plasma membranes, thus increasing glucose transport [61]. 

**Figure 3:**
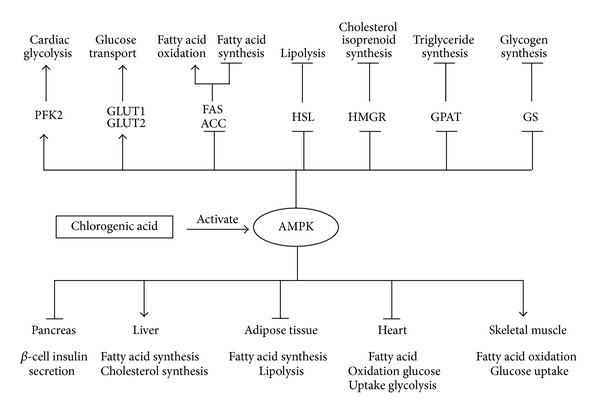
Chlorogenic acid regulates glucose and lipid metabolism via activating AMPK signal pathway.

Prabhakar and Doble [62] (2009) revealed that CGA stimulated glucose transport in myotubes via increasing expression of GLUT4 and PPAR-*γ* transcript. Subsequently, Ong et al. [63] (2012) investigated the role of CGA in the regulation of glucose transport in skeletal muscle isolated from *db*/*db* mice and L6 skeletal muscle cells. The results showed that CGA stimulated glucose transport in L6 myotubes in a dose- and time-dependent manner. In addition, it was demonstrated for the first time that CGA stimulates glucose transport in skeletal muscle via the activation of AMPK. In the following year, Ong et al. [18] further investigated the effects of CGA on glucose tolerance, insulin sensitivity, hepatic gluconeogenesis, lipid metabolism, and skeletal muscle glucose uptake in *Lepr* ^*db*/*db*^ mice. It was found that in *Lepr* ^*db*/*db*^ mice, acute treatment with CGA lowered AUC glucose in an OGTT. Chronic administration of CGA inhibited hepatic G-6-Pase expression and activity, attenuated hepatic steatosis, and improved lipid profiles and skeletal muscle glucose uptake, which in turn improved fasting glucose level, glucose tolerance, insulin sensitivity, and dyslipidemia in *Lepr* ^*db*/*db*^ mice. Furthermore the results of this study showed that CGA activated AMPK, leading to subsequent beneficial metabolic effects, such as suppression of hepatic glucose production and fatty acid synthesis. Inhibition and knockdown of AMPK abrogated these metabolic alterations. It suggested that CGA can improve glucose and lipid metabolism via the activation of AMPK (Figure 3). 

### 4.5. Inhibition of HMG CoA Reductase

 Gebhardt [64] demonstrated that CGA can indirectly yet efficiently inhibit *β*-hydroxy-*β*-methyl glutaric acyl coenzyme A reductase (HMG CoA reductase) in primary cultured rat hepatocytes and inhibit the synthesis of cholesterol. 

### 4.6. Strengthening the Activity of CPT

 Chlorogenic acid is able to strengthen the activity of carnitine palmitoyl transferase (CPT), a fatty acid oxidation speed limit enzyme, and promote the oxidation of fatty acid. This suggests a possible way for CGA involvement with lipid metabolism [65].

### 4.7. Inhibition of G-6-Pase Expression

In previous studies, many beneficial effects of CGA on the metabolism of glucose have been noted, with the possibility of improved systemic glucose control [66]. One of the dominant mechanisms is thought to be delayed absorption in the small intestine through the inhibition of glucose-6-phosphate translocase and reduction of the sodium gradient driven apical glucose transport [67].

Chlorogenic acid (CGA) has been shown to affect glucose metabolism [12, 34, 68, 69]. It has been shown to delay glucose absorption in the intestine through inhibition of G-6-pase translocase and reduction of the sodium gradient-driven apical glucose transport [68]. It was reported that CGA inhibited approximately 40% of glucose-6-phosphatase (G-6-Pase) activity in the microsomal fraction of hepatocytes [12]. Thus, CGA can decrease hepatic glucose output through inhibition of the activity of G-6-Pase [62, 66–68].

Chlorogenic acid (CGA) lowers the blood glucose concentrations and inhibits G-6-Pase, the two main metabolic pathways responsible for the release of glucose from the liver [36, 67, 70–72]. Previous experimental data shows that CGA promotes the uptake of glucose by liver cells and regulates the overproduction of glucose by inhibiting G-6-Pase; thereby, CGA controls glycemic status in T2DM patients [73]. In a 1997 study, Arion et al. [68] investigated the interactions of CGA and 2-hydroxy-5-nitrobenzaldehyde (HNB) with the components of the rat hepatic G-6-Pase system. Both CGA and HNB are competitive inhibitors of G-6-Pase hydrolysis in intact microsomes with Ki values of 0.26 mm and 0.22 mm, respectively. The authors revealed that CGA is the most specific T1 (the G-6-Pase transporter) inhibitor, and that CGA may selectively inhibit hepatic G-6-Pase, which is a rate-limiting enzyme involved in gluconeogenesis. 

Chlorogenic acid (CGA) is a novel insulin sensitizer that potentiates insulin action similar to the therapeutic action of metformin [30]. In contrast, CGA reduces blood glucose level by directly inhibiting G-6-Pase activity with the related effects of hepatic glycogenolysis [36] and gluconeogenesis [74]. Andrade-Cetto and Wiedenfeld [75] (2001) examined hypoglycemic effects of CGA in STZ-induced diabetic rats. No statistical difference between CGA and glyburide in the hypoglycemic effect after 3 hours was observed. The mechanism may be related to inhibition of glucose-6-phosphate displacement enzymes and glucose absorption. Wang et al. [69] (2012) investigated the effects of CGA on hepatic G-6-pase, skeletal muscle GLUT4 expression, blood glucose and lipid levels in STZ-induced diabetic rats. It was found that CGA exerted effects on improving blood glucose, TG, TC, insulin sensitivity, downregulating expression of G-6-pase and upregulating mRNA levels of GLUT4. Consequently the authors demonstrated that CGA may ameliorate the changes of glucose metabolism, lipid metabolism, insulin sensitivity, hepatic G-6-pase expression, and skeletal muscle GLUT4 expression in STZ-included SD diabetic rats. 

CGA has hypoglycemic and hypolipidemic functions, and can relieve the mouse insulin resistance development significantly by down-regulating the expression of G-6-Pase mRNA and up-regulating GLUT-4 transcript [42].

### 4.8. Upregulation of Expression of Hepatic PPAR-*α*

**Figure 4:**
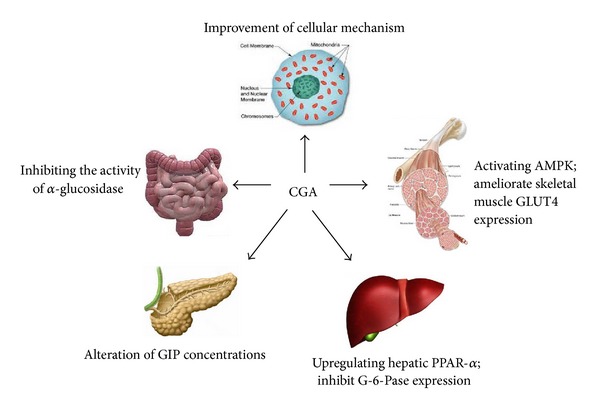
Chlorogenic acid's possible mechanism of action of regulating glucose and lipid metabolism.

 Zhang et al. [17] (2011) examined the effect of CGA on disordered glucose and lipid metabolism in *db*/*db* mice and its mechanism. They found that the mRNA expression level of G-6-Pase, the key enzyme that catalyzes the final step of glycogenolysis and gluconeogenesis, was significantly downregulated in db/db-CGA group when compared with db/db-CON group. Both the mRNA level and the protein expression levels of PPAR-*α* were significantly upregulated in db/db-CGA group compared with the db/db-CON group. The results of this study demonstrated that CGA improves the disordered glucose/lipid metabolism in *db*/*db* mice, which is speculated to be related with its role in modulating the adipokines secretion, upregulating expression of hepatic PPAR-*α*, and inhibiting expression of G-6-Pase (Figure 4).

Li et al. [76] (2009) investigated the effects of CGA on glucose and lipid metabolism under a high-dietary fat burden explored the possible role of peroxisome proliferator-activated receptor-alpha (PPAR-alpha) in these effects. It was found that CGA treatment significantly elevated the level of mRNA and protein expression in hepatic PPAR-*α*. The authors' results indicated that CGA may modify glucose and lipids metabolism, which may be attributed to PPAR-*α* facilitated lipid clearance in the liver and improved insulin sensitivity.

Wan et al. [19] (2013) investigated the hypocholesterolemic effects of the dietary consumption of CGA by monitoring plasma lipid profile in Sprague-Dawley rats. The authors found that CGA markedly altered the increased plasma total cholesterol and low-density lipoprotein but decreased HDL induced by a hypercholesterolemic diet with a dose-dependent improvement on both atherogenic index and cardiac risk factor. Lipid depositions in the liver were attenuated significantly in hypercholesterolemic animals supplemented with CGA. It is consequently postulated that hypocholesterolemic effect is the primary beneficial effect given by CGA, which leads to further secondary beneficial effects such as atheroscleroprotective, cardioprotective, and hepatoprotective functions. It suggested that the hypocholesterolemic functions of CGA are most likely due to the increase in fatty acid utilization in the liver via the upregulation of peroxisome proliferation-activated receptor *α* mRNA. 

Li [77] (2007) found that CGA can increase the activity of animals' hepatic lipase in the liver, the activity of PPAR*α*, *β*, and *γ* in the liver, and PPAR*γ* in visceral fat in various extents. PPARs are members of the nuclear receptor superfamily that play a key role in regulating glucolipid metabolism. It was demonstrated that CGA may regulate glucolipid metabolism by activating PPARs *in vivo* in rats. Thus, CGA may regulate glucolipid metabolism by a variety of mechanisms interactively. 

From previous as are stated, it has been testified and reported that CGA is able to exert vital roles on regulation of glucose and lipid metabolic disorders (Table 1), which are closely associated with the occurrence and progression of diabetes, obesity, hepatic steatosis, cardiovascular disease, and cancer. And increasing evidence shows that CGA will be exhibiting more potency in glucose and lipid metabolism in the near future. To our excitement, from diverse aspects, some mechanisms of actions of CGA are being elucidated, which will be beneficial to treat some diseases associated with glucose and lipid metabolic disorders.

## 5. Future Research Direction and Prospects

Present population epidemiological and animal studies suggest that CGA, which *in vivo* can regulate glucose and lipid metabolism and improves insulin sensitivity, may be capable of preventing and treating obesity, diabetes mellitus, and metabolic syndrome. However, this needs to be verified through the intervention studies of large-scale populations. Dose-response relationship and mechanisms of action of CGA's beneficial effects require further research and testification. Based on the study concerning the bioactivity of CGA and glucose and lipid metabolism, individuals may be guided to adopt a healthy diet, adjust dietary structure, and increase intake of natural plant ingredients, in order to prevent the occurrence and progression of chronic diseases. 

## 6. Conclusion

Accumulating research and studies, related with the role of CGA on glucose and lipid metabolism, have been conducted. While progress has been made, the mechanism on glucose and lipid metabolism has not yet been conclusively elucidated. The side effects of CGA have not yet been investigated comprehensively. Further research is required to elucidate both the short- and long-term effects of CGA on glucose and lipid metabolism. It is hoped that research concerning the mechanism of action of CGA on glucose and lipid metabolism will be developed in the future, and that information on the potential clinical applications of CGA will increase.

## Figures and Tables

**Table 1:** Summary of studies of CGA on glucose and lipid metabolism.

Study (references)	Year	Animal experiment			Clinical trial	Findings	Mechanism of action
		*In vivo*/ *in vitro *	Animal/cell	Disease model			
Karthikesan et al. [31]	2010	*In vivo *	STZ-NA-induced diabetic rats	T2DM	—	Hypoglycemic and antidiabetic effects	—
Karthikesan et al. [32]	2010	*In vivo *	STZ-NA-induced T2DM adult Wistar rats	T2DM	—	hypoglycemic and antidiabetic effects	—
Pari et al. [33]	2010	*In vivo *	STZ-NA-induced T2DM adult Wistar rats	T2DM	—	hypoglycemic and antidiabetic effects	—
Bassoli et al. [12]	2008	*In vitro *	Liver perfusion	—	—	Reduction in the plasma glucose peak in the oral glucose tolerance test	—
Alonso-Castro et al. [34]	2008	*In vitro *	3T3-F442A murine adipocytes		—	Exerting antidiabetic effects on stimulating glucose uptake in both insulin-sensitive and insulin-resistant adipocytes	—
van Dijk et al. [35]	2009	—	—	—	Overweight men	Reducing early fasting glucose and insulin responses in overweight men during an OGTT	—
Ahrens and Thompson [36]	2013	—	—	—	T2DM patients	Lowering the glycemic impact of foods and lowering background blood glucose levels of T2DM	—
Tousch et al. [37]	2008	*In vitro *	L6 muscular cells		—	Stimulation of insulin secretion	—
Johnston et al. [38]	2003	—	—	—	Healthy fasted volunteers	Stimulation of insulin secretion	—
Liang et al. [42]	2013	*In vivo *	Mouse induced by high fat emulsion	Insulin resistance	—	Improvement of glucose tolerance and insulin resistance	—
Rodriguez de Sotillo and Hadley [14]	2002	*In vivo *	(fa/fa) Zucker rats	T2DM	—	Lowering serum and hepatic CG and TG levels	—
Shimoda et al. [46]	2006	*In vivo *	Male ddy mice	—	—	Inhibiting fat absorption and activating fat metabolism in the liver	—
Cho et al. [47]	2010	*In vivo *	High-fat diet induced-obese mice	T2DM	—	Improvement of obesity-related hormones levels	—
Karthikesan et al. [48]	2010	*In vivo *	STZ-NA induced diabetic rats	T2DM	—	Alterations in lipids, lipoproteins, and enzymes involved in lipid metabolism	—
Wang et al. [50]	2012	*In vivo *	Mice induced by high-fat diet	T2DM	—	Improvement of antioxidant activities	—
Rodriguez de Sotillo et al. [15]	2006	*In vivo *	Obese Zucker (fa/fa) rats	T2DM	—	Improvement of glucose tolerance and mineral pool distribution	Improvement of cellular mechanisms
Zheng et al. [52]	2007	*In vivo *	Kud:Wistar rats	—	—	Reducing the postprandialblood glucose concentration	Inhibiting the activity of *α*-glucosidase
Tunnicliffe et al. [59]	2011	*In vivo *	Male Sprague-Dawley rats	T2DM	—	Attenuating AUC for blood glucose	Alteration of GIP concentrations
Ong et al. [63]	2012	*In vivo* *In vitro *	db/db mice L6 skeletal muscle cells	T2DM	—	Stimulating glucose transport in L6 myotubes in a dose- and time-dependent manner	Activation of AMPK
Ong et al. [18]	2013	*In vivo* *In vitro *	Lepr db/db mice Hepatoma HepG2	T2DM	—	Attenuating hepatic steatosis, improving lipid profiles and skeletal muscle glucose uptake, glucose tolerance, insulin sensitivity, and dyslipidemia	Activation of AMPK
Gebhardt [64]	1998	*In vitro *	Primary cultured rat hepatocytes	—	—	—	Inhibiting HMG CoA reductase and inhibiting the synthesis of cholesterol
Frank et al. [65]	2003	*In vivo *	Sprague-Dawley rats	—	—	—	Strengthening the activity of carnitine palmitoyl transferase (CPT)
Arion et al. [68]	1997	*In vitro *	Rat hepatic cells	—	—	—	CGA is the most specific T1 (the G-6-Pase transporter) inhibitor, and may inhibit hepatic G-6-Pase
Wang et al. [69]	2012	*In vivo *	Chemical-induced diabetic rats	T2DM	—	Exerting effects on improving blood glucose, TG, TC, and insulin sensitivity	Downregulating expression of G-6-pase and upregulating mRNA levels of GLUT4
Zhang et al. [17]	2011	*In vivo *	db/db mice	T2DM	—	Improvement of the disordered glucose/lipid metabolism	Upregulating expression of hepatic PPAR-*α*
Li et al. [76]	2009	*In vivo *	Golden hamsters fed on high-fat diet	T2DM	—	Modifying glucose and lipids metabolism	Upregulating expression of hepatic PPAR-*α*
Wan et al. [19]	2013	*In vivo *	Sprague-Dawley rats induced with a high-cholesterol diet	Hyperlipidemia	—	Altering the increased plasma total cholesterol and low-density lipoprotein but decreased HDL induced by a hypercholesterolemic diet with a dose-dependent improvement	Upregulating expression of hepatic PPAR-*α*

STZ: streptozotocin; NA: nicotinamide; CPT: carnitine palmitoyl transferase; HMG CoA reductase: *β*-hydroxy-*β*-methyl glutaric acyl coenzyme A reductase.

## Abbreviations

AUC: Areas under the curve
AOM: Azoxymethane
b.w.: Body weight
CaA: Caffeic acid
CGA: Chlorogenic acid
CH: Cholesterol
CRA: Chicoric acid
FFA: Free fatty acid
GCE: Green coffee bean extract
G-6-PASE: Glucose-6-phosphatase
GIP: Glucose-dependent insulinotropic peptide
HBV: Hepatitis B virus
HDL: High-density lipoprotein
HHQ: Hydroxyhydroquinone
HOMA-IR: Homeostasis model assessment for insulin resistance
MAPKs: Mitogen-activated protein kinases
MMP: Matrix metalloproteinase
NA: Nicotinamide
NF-kappaB: Nuclear transcription factor *κ*B
ROS: Reactive oxygen species
STZ: Streptozotocin
TC: Total cholesterol
TG: Triacylglycerols
T2DM: Type 2 diabetes mellitus
THC: Tetrahydrocurcumin
TZD: Thiazolidinedione.
